# Hospital and Institutional News

**Published:** 1916-11-04

**Authors:** 


### HOSPITAL AND INSTITUTIONAL NEWS.

### ONE OF FLORENCE NIGHTINGALE'S DOCTORS.

A venerable figure disappears from Hemel Hempstead with the death of Dr. James Vaughan-Hughes, a Crimean colleague of Miss Nightingale, at the advanced age of ninety-five. When serving under Sir John Hall, Chief of the Medical Staff in the Crimea, he was for a time in medical charge at Balaklava Hospital, and attended Florence Nightingale when she herself was struck down by fever. On her recovery he borrowed Lord Dudley's yacht to convey her back to Scutari. Later on, the relation of doctor and patient was reversed; for Dr. Vaughan-Hughes himself got cholera, and was nursed by Miss Nightingale and her staff. In spite of his age, Dr. Vaughan-Hughes was wonderfully active until quite recently; he could and did ride on horseback, drive his own pair, and even his own motor-car up to a few months ago. He was also in the habit of wearing on his ordinary clothes the medals and decorations won in earlier days; so it is readily understandable that Hemel Hempstead is the poorer by a personality at once vigorous and venerable.

### MEMORIAL TO AN IRISH SURGEON.

The hall of the Royal Victoria Eye and Ear Hospital is now decorated with a memorial to the late Sir Henry R. Swanzy, who was President of the Royal College of Surgeons in Ireland from 1906-08 and former President of the Ophthalmological Society of the United Kingdom. Well known for his eye work, in connection with which he issued many publications, it is natural that the Royal Victoria Eye and Ear Hospital should wish to perpetuate his connection with it by a personal tribute to his memory. For this purpose, a plaque bearing Sir Henry Swanzy's portrait in relief, with an inscription on a panel beneath, has now been erected. It has lately been fashionable to look somewhat askance on these "æsthetic memorials" (to use a generic term which implies no necessary tribute to their artistic value), unless associated with some fund or extension directly connected with the hospital's work. We have always challenged this so-called common-sense, practical way of regarding memorials, on the ground that a hospital lives in the best sense largely by tradition and the great names associated with it, and that therefore it is highly important for these names to be conspicuous by their presence in bust and picture, so that the board-room or hall shall become in time a gallery, in which every man of honour and authority associated with the hospital will have his niche. Tradition requires the outward sign as well as the inward influence, and the outward sign can only be given in works of art, whose sole use and object is to embody that, and that only.

### HOSPITAL ARCHITECTURE.

There can be no doubt that the general architectural character of our hospital building is either deadly dull or replete with meretricious ornamental exuberance, leaving much to be desired. As public buildings hospitals should bear a civic impress, and a leaning towards the monumental in part. On the Continent, in France, and particularly in Germany, external architectural expression has been allowed to run riot. Fitness of purpose and beauty of proportion, resulting in part from the concentration of definite architectural features, should be diligently sought for, and can be secured without undue extravagance in outlay. We are glad again to call attention to this important matter in an article by Mr. Pite, which will be found on p. 89. Our readers may recall a note on this very subject which appeared in this journal for February 19, 1910, page 606. The point there emphasised may well be repeated again here: "It is of the highest importance to the community that its hospitals should impress the beholder, and to impress it should possess beauty as well as dignity"; or as Tenon has so well put it: "Les hôpitaux sont la mesure de la civilisation d'un peuple."

### MILITARY HOSPITAL CONSTRUCTION.

In another column (p. 107) will be found a letter from Professor Simpson, setting forth his opinion on some recent hospital construction at Leicester. Professor Simpson, as our readers will see, is emphatic in his preference for the open wards built to local designs over the closed wards erected there by the Barrack Department of the War Office. There is no need to labour the points which Professor Simpson makes, for they are ably and amply explained in his letter. But we do trust that the authorities will take his words to heart, and that no more "pre-Crimean" buildings will be sanctioned or erected by any Government department.

### A MEDICAL MISSIONARY EXHIBITION.

The one branch of medicine of which least is known to the public, and but little even to the profession and to hospital men, is that of medical missions. Here and there an enthusiast exists, outside the ranks of those engaged in it, but such enthusiasts are far between and have never yet succeeded in leavening the lump of public ignorance concerning an important and most anxious side of hospital and medical work. The idea, therefore, of holding a public exhibition illustrating the varied work carried on in remote parts of the world, among people of strange language and curious customs, is one that well might be extended. Such an exhibition has lately been held at Newcastle under the auspices of the "S.P.G.," but we should like to see one in London entirely conducted by one of the medical missionary bodies. The ideal, as we have often pointed out, is for every missionary to receive a medical training, and meanwhile everything which can familiarise the public with the invaluable work of the medical missionary is to be welcomed. There is no better way than the holding of medical missionary exhibitions.

### A SCATHING REPORT.

It must be some years since such sweeping condemnation of any institution for the insane was pronounced officially as that contained in the report of the inquiry by the Scottish Board of Control into the medical management of the Gowrie House Private Asylum, Dundee. It would seem that the directors of the Royal Asylum, who maintain Gowrie House, formally complained of irregularities there, and asked for an inquiry. The result of this inquiry is published in the form of a letter addressed to the Local Board of Control, which is stated to be the Parish Council. The *Dundee Advertiser* has it that the Local Board has wilfully shut its eyes to abuses which were notorious and patent, and has some very hard words upon the "smug self-sufficiency" of its members. The editor also asks whether all is well at West Green Asylum, which is under the same medical superintendent and in the same ring fence. If the facts are as he sets them forth, it is difficult to imagine that the medical officer in question will be retained at either institution, no matter how many friends he may or may not have on the Parish Council.

### THE BOARD OF CONTROL'S SEVERE CENSURE.

The passages in the report which touch the root of the matter can be briefly indicated. The Board of Control holds that the evidence discloses a state of affairs which amply justifies the dissatisfaction of the Royal Asylum directors. Throughout the period from the end of February to August 8, 1916, the medical superintendent is held to have acted with a "flagrant disregard of his duties"; and in particular it is considered that in each of the four specific cases investigated he was "guilty of gross neglect of his duty to these patients and is deserving of severe censure." The Board is of opinion that without delay effectual steps must be taken, however drastic, to prevent any recurrence of similar irregularities in the future; and requires that the patients in Gowrie House shall have the advantage of regular daily visits from a medical man. The work of the officials other than the medical superintendent is not included in these strictures; indeed, that of the matron is highly praised. There can be, we should imagine, only one course for the Local Board to follow, especially as the Board of Control desire to be informed what action is taken as the result of the inquiry. The editor of the *Dundee Advertiser* evidently believes that the force of an indignant public opinion can alone prevail over the supineness of the Parish Council; but we fancy that body will bow to the Board of Control, unless their Scottish obstinacy is unaccompanied by its usual complement of Scottish caution.

### THE NORTH WALES ASYLUM AND THE BOARD OF CONTROL.

The Committee of Visitors of the North Wales Asylum is up in arms against the action of the Board of Control for England and Wales in vetoing the proposal to board out chronic cases from the Denbigh Asylum at the Llanfyllin Union Workhouse; and some very heated speeches on the subject were made at the last quarterly meeting of the committee. It is naturally out of the question for anyone who has not visited the Llanfyllin Workhouse, and has not studied the details of the scheme which the Board of Control has rejected (after sending a Commissioner to inspect the workhouse), to pronounce an opinion as to whether the Board or the Committee of Visitors has more abstract right upon its side. But it is quite clear to anyone who reads the debate at Denbigh, as reported in the *Liverpool Post*, that there are some very injudicious and hot-headed persons on the Committee of Visitors of the North Wales Asylum. One after another of them got up and abused the Board of Control up hill and down dale, in unmeasured language. Whether any members of the committee kept their tempers the report does not disclose; none of those who spoke seems to have succeeded in doing so. Very seriously we suggest to the North Wales Asylum Committee that it is improbable that the members of the Board of Control are the idle and incompetent people that the speakers at Denbigh represented them to be; and that to hurl fiery abuse at the heads of a Government Department merely because they take views different from those favoured locally is a form of kicking against the pricks which is singularly unlikely to prove profitable.

### RETIREMENT OF A MENTAL HOSPITAL SUPERINTENDENT.

On Thursday, November 9, Dr. W. H. Bowes, medical superintendent of the Blackadon Lunatic Asylum, Plymouth, lays down the responsibilities of the post which he has occupied for twenty years. His resignation involves a great loss to the community, for the value of his able services, extending over such a long period of years, cannot be adequately reckoned. The Asylum Visiting Committee has placed on record its high appreciation of Dr. Bowes' work, and has computed and settled his retiring allowance at £325 per annum.

### SERIOUS HOSPITAL FIRE IN CANADA.

On October 26 a fire, which is attributed to a defective chimney, destroyed the great Elizabeth Hospital at Farnham, Quebec. In all there were three hundred and fifty human beings on the premises at the time of the outbreak, and the fire spread so rapidly that most of them were obliged to jump out of the windows in order to effect their escape. Twenty-five of the children were injured in attempting to escape thus by jumping from the third storey. It is reported that five children are dead and fifteen others missing. This serious death-roll and list of injured proves how sudden and disastrous the fire must have been; details are not yet to hand by mail as to whether the destruction of the buildings is total, or whether parts of them were saved from the fire sufficient to form the nucleus of a rebuilding scheme.

### A WELSH ORTHOPÆDIC HOSPITAL.

Colonel Lynn Thomas, C.B., to whose initiative and enthusiasm the Welsh Orthopædic Hospital, soon to be established at Cardiff, will be a permanent memorial, is determined that the institution shall be equipped on the most modern lines and rank as equal to the best in the country. It is in this spirit that he and the others associated with him in the project have got to work. The intention is to make the hospital the centre for orthopædic treatment in the Principality. Not only will the disabled sailors and soldiers of Wales be there treated and fitted with artificial limbs, manufactured on the spot, but in the old Mansion House or its adjoining buildings they will receive technical training in the art or craft to which they are best suited individually. The needs of maimed junior officers will also be met by the hospital. The Prince of Wales has shown his interest in the institution which is to serve the soldiers from his Principality by signifying, in a letter received by the Lord Mayor of Cardiff, his consent to the hospital being named after him.

### SANATORIA AFTER THE WAR.

The future of the temporary hospitals which have been erected for military hospitals is full of interest; and, apart from the influence which they may be expected to exercise on hospital architecture, a definite place may be found for many of the structures themselves. This should be especially the case with the open-air wards that now exist in various parts of the country, and we note that the new open-air pavilion which has now been added to the Northumberland War Hospital, thanks to the generosity of the Elswick and Scotswood workers, is destined for use as a sanatorium when the war is over. If this plan can be carried out here and at other centres something should be done to provide for the shortage of sanatorium beds, which is still great, especially in respect of advanced cases. That is a prospect upon which some hopes may be built, for the buildings which the war has created must not be wasted when the soldiers require them no longer.

### THE RISE OF INSTITUTIONAL SALARIES.

Asylum committees all over the country are finding it necessary to increase the salaries, wages, or allowances of almost every grade of worker in their employment. A typical instance of these all-round advances is provided by the Plymouth Lunatic Asylum. Here the pay of the night nurses has been increased to £24 per annum, rising by annual increments to £39. The wages of the cook and laundress have been increased by £5 per annum, and those of the needle-mistress, laundry maids, and kitchen maids to £30. The male attendants have been granted a war bonus of 2s. a week, and a similar weekly bonus is now being paid to the upholsterer, blacksmith, painter, and tailor engaged at the asylum.

### GREAT EFFORT BY A HIGH SHERIFF.

Philanthropic effort and the shrievalty are not so often associated as to make the record of Leicestershire's High Sheriff uninteresting. Impressed by the need of increasing the income of the Royal Infirmary by several thousand pounds per annum, Mr. Alfred Corah, J.P., has used his office to considerable effect and purpose in a personal appeal to business houses of the town and county. The success of this appeal is shown in a list of new and increased annual subscriptions which he has recently announced. It was based upon three special points: (1) The satisfactory financial position of the principal industries; (2) the high cost of living, which seriously affects the voluntary institutions; (3) the need for avoiding the constant appeals for funds for extension purposes, which, Mr. Corah submitted, could be largely provided for in the future if the institution's income were sufficient to allow the setting aside of an annual sum for future contingencies. The response was generous. Fifty-six firms and companies became new or increased annual subscribers of 50 guineas each, and forty-four of 25 guineas each. Other firms became donors, and others became subscribers of lesser amounts. Altogether Mr. Corah was able to announce a first list of contributions amounting to upwards of £4,300.

### A GOOD FAMILY RECORD.

In a great industrial centre like Leicester, with its thriving industries, it is pleasant to observe the great interest evinced in the local hospital, which has for many years shown a splendid record of progress for an institution which cannot claim the impetus invariably given by a medical school. Proof of this progress is seen from an advertisement which has recently appeared in The Hospital inviting applications for a pathologist at a salary of £500 per annum. This, coming so closely upon the announcement that the money has been raised for the building of a modern pathological department, so soon as opportunity offers for it to be constructed, shows that the governors are wide awake to the importance of the efficiency of the institution and to the future of scientific research. We congratulate Mr. Alfred Corah on his splendid service to the county and to the Royal Infirmary. Mr. Corah is associated with a firm which has been one of the most generous of the institution's supporters. It is largely due to the firm's employees that the Saturday Hospital movement in Leicester was established on systematic lines. Many years ago the employees commenced to contribute a penny weekly to the charitable institutions of the town. Their contribution to the infirmary last year reached the substantial figure of £550. Mr. Alfred Corah's office as High Sheriff makes him an *ex-officio* member of the board of governors of the infirmary. His son, Mr. J. H. Corah, is also a member of the board, and his elder brother, Mr. J. A. Corah, likewise a member, has just announced his intention to present to the institution a recreation-room for the wounded soldiers undergoing treatment therein. It is of the utmost encouragement to find families in this way associating themselves with philanthropic endeavour —the "Corah" family are evidently very interested and very proud of their local hospital.

### A STATE MEDICAL SERVICE.

A good deal of excitement has been manifest lately amongst those medical men—an almost infinitesimal minority, by the way—who take an interest in medical politics and economics, by various suggestions that a State Medical Service may be a feasible proposition after the war. An anonymous "medical correspondent" of the *Times* has been ventilating the idea in a rather vague kind of way, and has at any rate stimulated both friend and foe into articulate activity. The writer in the *Times* hardly displays the grasp of current medical opinion for which Sir J. Mackenzie Davidson was notable when he guided editorial pronouncements in that newspaper; but he makes one very sensible remark when he says that doctors should define what they mean by a State Medical Service before they quarrel about it. He goes on further to suggest that the Medical Service of the Army, which is a form of State Service controlled by doctors, may be taken as a model for a civil State Service; that is, for a Service which the medical profession is responsible for managing and controlling. It is all rather a tentative essay in medical politics; but meanwhile the enthusiasts on either side are keeping the ball rolling merrily.

### FOR AND AGAINST.

First of all, the conference of panel practitioners, held the other day under the auspices of the Insurance Commissioners, passed unanimously a resolution affirming the strongest opposition to a State Medical Service; and the Secretary of the B.M.A. at once communicated this to the *Times*, with special reference to that newspaper's editorial coquetting with the notion of such a Service. The weakness of the position of the gentlemen who passed that resolution is that they are one and all engaged in a half-way house to State Service, that they are men who have stayed snug at home, and that they cannot be exactly described as the intellectual cream of the profession: all of which things have been duly rubbed in by some of their opponents. Next came a protest from a temporary captain in the R.A.M.C., who raised a very important point in reference to the ruined practices of the thousands of Territorial and Special Reserve medical officers on active service. Whether his statement is true that "many thousands" of the temporarily commissioned would welcome a State Medical Service at the end of the war is doubtless a matter of opinion. But this problem of deteriorated practices is a very serious one and has not received half the attention it deserves. One way and another it is clear that opinion is much divided, and that new currents of thought have been put in circulation.

### THE TRADE IN ABORTIFACIENTS.

It is stated that some degree of restriction, albeit only a slight one, is to be placed upon the sale of lead in a form that lends itself to use as an abortifacient. It is understood that lump diachylon is to be added to Schedule 1 of the Poisons and Pharmacy Act, 1908. The result of this will be that only qualified chemists will be able to sell it, and then only under certain restricted conditions. Unauthorised druggists, herbalists, and quacks will be prohibited, under penalty, from dealings in this compound of lead, which is extensively used, especially in the Midlands, for the purpose of procuring abortion. It cannot be too widely made known that salts of lead are powerful and deadly irritant poisons; and that the misguided women who take doses of them sufficient to procure abortion upon themselves succeed in their object only when they take enough to cause definite (and not infrequently fatal) symptoms of lead poisoning. The action now contemplated is good as far as it goes; but we look for much greater extension of the principle in the future.

### THE SUNDERING CINEMA.

The question of hospitals accepting sums from cinematograph entertainments held on Sunday nominally on their behalf is again engaging attention. The matter has been discussed by the Bishop of Willesden in connection with the refusal of the Hampstead General and North-West London Hospital to accept an offer of £27 for each Sunday entertainment at the West-End Cinema in Coventry Street. His own view, as given in the *Times*, was this: "The whole question will have to come before the Hospital Sunday Fund, and I hope that hospitals will have to choose between help from that Fund and help from the Sunday cinemas—unless the London County Council can be persuaded at their coming sessions to withdraw permission for Sunday entertainments, as the Middlesex County Council has done." This statement is worth quoting because it once more illustrates our contention that the attitude of the hospitals towards these Sunday entertainments must be guided not merely by any financial gain that may accrue to them at the moment, but by their responsibility towards the other hospitals, which must not be exposed to the injury that would result from any dislocation of the Sunday Fund. It is not fair or right to imperil the stability of that Fund by any selfish policy; and this being so, no hospital should have any traffic with these cinema shows, so long as acute controversy is excited by them. Experience has proved this, and the Bishop of Willesden's statement is a useful reminder of it.

### THE ANOMALOUS POSITION OF THE MILITARY CHAPLAIN.

A plea recently made in the *Church Times* that additional scope should be given to the chaplains of military hospitals by the commandants, who, it is complained, are often bound by red-tape, raises the question as to the duties of our military chaplains. The point of our contemporary's plea appears to rest on the duty of the chaplain, so far as he can, to raise the tone of the entertainments given to the wounded, and it is urged that on these lines the chaplains "have distinct chances of social service," especially since, "properly regarded, their work goes far beyond the holding of services." This latter statement is no doubt true, and it seems ungracious perhaps to suggest that, in spite of the services and decorations which the chaplains have rendered and received, yet the position of a chaplain remains somewhat an ill-defined, if not anomalous one. While the *Church Times* suggests that the work of the chaplains should go beyond the limits of holding services and ministering to the spiritual needs of individual men, implying that these duties are primary and but the letter of the law, more than one chaplain has been understood to complain that he seems to be expected to do nothing but organise concert parties, be the *major-domo* of the entertainments, and the good fellow of the recreation-room. If this be so, the complaint of the *Church Times* should be directed rather against the excessive preoccupation with lay matters that is forced upon the chaplains of the English Church. A significant fact, at all events, in support of such a view is that so much appears to depend upon the character of the individual chaplain and so little on his status and the duties of his office. No doubt the Church of England is generally stronger in her practice than her theory, and perhaps wisely so; but in war the individual is apt to be sacrificed thereby, and the anomalous position of Church of England chaplains is a case in point.

### A SURCHARGE REMITTED.

The Camberwell Board of Guardians have had considerable difficulty in inducing the Local Government Board to remit a surcharge of £54 17s. paid to Dr. W. J. Keats, the late medical superintendent at their infirmary. The Guardians originally desired to increase his salary by £100 a year, but the Local Government Board persistently declined to sanction any increase beyond £50, whereupon the Guardians resolved to pay Dr. Keats £75 for preparing certain reports relating to old-age pensioners admitted to the infirmary, and also for extra work under the National Insurance Act. The district auditor took objection to these payments, maintaining that Dr. Keats was not subjected to any additional work inasmuch as he had the reports in his possession as chief officer of the infirmary. It was clear to him that the grant was made immediately after the Local Government Board had refused to sanction the second increase of £50, and was done in contravention of the Board's decision. The payments were, therefore, illegal. The Local Government Board upheld the decision of their district auditor, adding that in view of the Guardians' action they had grave doubts as to whether they ought to remit the surcharge, but as the payments had ceased immediately after the district auditor's decision they would relieve the Guardians of their liability and cancel the surcharge.

### JUVENILE EMPLOYMENT.

The thirteenth Memorandum issued by the Health of Munition Workers Committee deals with the important topic of juvenile employment in war industries. The development of munition works has entailed not merely a large amount of employment of boys and girls, but has also provided them with much higher wages than such young people have ever been able to earn before. Although such high wages are, in the Committee's opinion, beneficial to health to the extent that they have brought good food and suitable clothing within reach of all, on the other hand there is grave reason to fear that they lead also to self indulgence and extravagance, and even to gambling, thriftlessness, and immorality. In this connection the formation of War Savings Associations is an obvious step in the right direction, although the Committee do not mention it. Equally important is it that such Associations shall be run on simple lines, be under the direct observation of the Welfare Supervisor, and that elected delegates of the employees themselves shall take a small share in the management. The Committee rightly lay great stress on the especial value of "Welfare Supervision" (already the subject of the Committee's second Memorandum) when the employees are youths or young women. In this Memorandum they lay down an outline of the duties of the "Boy Visitors" in the scheme of supervision for boys which has recently been initiated at the Royal Arsenal Factories at Woolwich. Stress is also laid on the importance of recreation-grounds, clubs, swimming-baths, and country camps in summer.

### PROMISING THE IMPOSSIBLE.

The Oklahoma State Board of Medical Examiners, says the *Medical Record* of New York, has revoked a physician's licence for undertaking, for a fee, to cure an incurable disease. It appeared, on appeal to the Oklahoma Supreme Court, that a document headed "Absolute Guaranty" had been handed to the patient, in which the defendant agreed to refund all moneys paid should a complete cure not be afforded by the treatment, and the patient agreed to follow the defendant's directions through a period sufficient as deemed by the defendant to effect a complete cure, failing his following the directions so given the agreement to become null and void. The defendant claimed this was not a guaranty of cure, but only a guaranty to refund the fee in the event of the treatment proving unsuccessful. The appeal failed, the Court considering that an assurance of a permanent cure had been held out to the patient.

### ALLEGED HOSPITAL ABUSE IN NEW ZEALAND.

The medical profession of New Zealand, as voiced by a leading article in the *Medical Journal of Australia*, consider that unfair competition is caused by the admission of well-to-do patients to Launceston Hospital, especially when these persons pay operation as well as maintenance fees. It is pointed out that, with the exception of a very few beds under the care of a small honorary staff, most of the patients were attended by the medical superintendent, who had it within his power to admit all surgical cases into his own wards and to draft off the less interesting cases to the visiting staff. The Council of the Tasmanian Branch of the British Medical Association have sought to improve matters by directing the attention of the Chief Secretary to the disadvantageous position in which the local practitioners find themselves. This representation was passed on to the hospital board for their comments, and the general feeling of that body was that as long as patients paid according to their means, the hospital should be available to the whole community, although the rights of practitioners were so far recognised by the admission that doctors in practice in the city should be permitted to perform operations on their own patients in the hospital. The Minister's reply to the Medical Association has not yet been published.

### THE WELSH NATIONAL MEMORIAL ASSOCIATION.

It is announced in the *London Gazette* that the Welsh National Memorial Association has presented a petition to the King in Council praying for the grant of a supplemental charter. The petition has been referred to a committee of the Lords of the Council. All petitions for or against the grant of a supplemental charter should be sent to the Privy Council Office on or before November 7.

### THE PRAIRIE NURSE.

District nursing in the remoter parts of the Dominions (and indeed among the wild parts of the Highlands and Islands of Scotland) presents many difficulties, among which is the scarcity of candidates for such lonely work as this. The Colonial Nursing Association, however, hopes to extend the work already accomplished by inducing the widows of officers and non-commissioned officers to become trained midwives, and then to agree to undertake a two years' tour of service as mothers' nurses. The idea is to begin with Canada, and, while the nurses will be placed under the charge of local practitioners, it is hoped that the scheme may offer a new profession and a field of service to women whose homes have been broken up by the war.

### MILITARY HOSPITAL EXPANSION AT CARLISLE.

A new military hospital of 640 beds is to be formed at Carlisle. Fusehill Workhouse and Shap Workhouse, as well as schools at Newtown and Brook Street, are to be used as premises for this hospital. The Fusehill Workhouse alone provides 400 beds; the 200 present occupants will be provided for elsewhere, and a commendable spirit of helpful co-operation amongst the boards of neighbouring Unions is being manifested in this respect. The County Director (Mr. Gandy) states that this hospital will be run on somewhat novel lines—that is, it will neither be wholly military nor wholly voluntary in its management and staffing. The officer in charge will be known as the Commandant, not as Officer Commanding: he will, however, be a lieutenant-colonel of the R.A.M.C., and he will bring with him two R.A.M.C. officers and sixty trained nurses. About forty V.A.D.s are required as well, and it is hoped that this number, properly instructed and certificated, can be provided in Carlisle; otherwise there will have to be an importation, and this introduces problems of billeting, etc., which will be avoided if the local supply proves satisfactory. The new hospital will be under the control of the hospital base at Carlisle, but it is not yet settled whether it will be directly so controlled, or through the intermediary command of Fazakerley.

### DEATH OF A HOSPITAL TREASURER.

The borough of Leicester has lost one of its foremost citizens and the Leicester Royal Infirmary its Treasurer in the death of Sir William Wilkins Vincent, J.P. He was twice Mayor of the borough, the second time in the Coronation year 1910. For many years he was Chairman of the Finance Committee of the Town Council, and was largely associated with the Derwent Valley Water Board scheme, which secured an adequate water supply to the borough. For several years he was Chairman of this undertaking. He served for some years as Chairman of the Finance Committee of the Infirmary, and on the death of Mr. S. F. Stone was elected Treasurer of the institution under a new rule which made the post tenable for a period of three years. He was also elected a Trustee of the institution. His life was spent largely in the service of the town and its institutions. He died in his seventy-fourth year, leaving one son and two daughters, one of whom is married to a well-known medical practitioner in the borough.

### TWO NEW MEDICAL V.C.s.

Two out of the fifteen Victoria Crosses awarded last week are conferred upon captains who were members of hospital medical staffs when the war broke out. Captain William B. Allen, after taking his M.B. and Ch.B. at Sheffield University with honours, joined the Royal Hospital staff at Sheffield about the middle of 1914, but threw up his post a few days after war was declared to become a lieutenant in the West Yorkshire Field Ambulance. It was only in August of this year that Captain Allen was awarded the Military Cross for gallantry in the field. The other medical V.C. is Captain Noel G. Chavasse, second son of the Bishop of Liverpool, who was house physician and surgeon at the Southern Hospital, Liverpool, from 1912 until he became medical officer to a Liverpool battalion. Captain Chavasse, who is thirty-one, was awarded the Military Cross last year for conspicuous service in collecting wounded after a charge.

### THIS WEEK'S DRUG MARKET.

While the general tendency of prices is in a downward direction, several drugs are dearer for the time being. In spite of the large stocks of ipecacuanha prices have not receded to a very substantial extent; but a further fall in value seems inevitable. Both citric acid and tartaric acid are again rather lower. The price of balsam Peru has a downward tendency. Cubebs are scarcer and dearer. The value of castor oil is firmly maintained. Scammony resin is dearer. Among synthetic products, salicylic acid, sodium salicylate, hexamine, salol, phenazol, methyl salicylate and acetyl-salicylic acid all have a downward tendency in price. There seems to be no prospect of any considerable decline in the price of cod-liver oil; indeed, in view of the fact that the chief consuming season is now beginning, prices may become still higher. Bromides are unchanged.

### TO OUR READERS.

**Contributions are specially invited from any of our readers to these columns. They should deal with topical subjects and news. They must be authenticated for the information of the Editor only. The minimum payment if published is 5s. There is no hard-and-fast rule as to space, but notes of about twenty lines in length are preferred.**

